# Psychological Capital and Burnout in Teachers: The Mediating Role of Flourishing

**DOI:** 10.3390/ijerph17228403

**Published:** 2020-11-13

**Authors:** Carlos Freire, María del Mar Ferradás, Alba García-Bértoa, José Carlos Núñez, Susana Rodríguez, Isabel Piñeiro

**Affiliations:** 1 Department of Psychology, University of A Coruña, 15071 A Coruña, Spain; alba.gbertoa@udc.es (A.G.-B.); susana.rodriguez1@udc.es (S.R.); isabel.pineiro.aguin@udc.es (I.P.); 2 Faculty of Psychology, University of Oviedo, 33003 Oviedo, Asturias, Spain; jcarlosn@uniovi.es

**Keywords:** psychological capital, flourishing, burnout, occupational health, teaching

## Abstract

In keeping with the growth in the development of healthy environments in organizational contexts, in recent years, there has also been increasing interest in the identification of personal psychological resources that contribute to improved worker mental health. From this proactive approach, this study examines the mediating role of flourishing in the relationship between psychological capital (PsyCap) and burnout in teachers, a professional group that is particularly prone to suffering from this syndrome. A total of 1379 teachers from pre-school, primary, secondary, and vocational education systems participated in the study. The mediating effect of flourishing was determined via mediation analysis using the PROCESS macro. The results showed that flourishing partially mediates the negative effect of PsyCap on the three symptoms of burnout (emotional exhaustion, depersonalization, and lack of professional accomplishment). These findings indicate that both PsyCap and flourishing may be effective personal resources in reducing teacher burnout. Therefore, in order to prevent burnout, it is advisable to design interventions that combine PsyCap and flourishing.

## 1. Introduction

The development of workplaces with healthy atmospheres and the preoccupation of workers’ well-being constitute one of the key strategies of the United Nations 2030 Agenda for Sustainable Development [1] with the aim of promoting social progress and the growth of individuals, families, and communities. This emerging approach therefore places workers’ well-being as a cornerstone in the achievement of healthy, effective, and productive occupational life [2,3], and makes it a key factor for public health [4].

The emphasis on aspects linked to optimal positive functioning in the organizational environment is anchored in positive psychology, a discipline that arose at the beginnings of this century with the aim of identifying and encouraging the conditions that contribute to individual, group, and institutional flourishing [5]. Flourishing would thus represent the true gold standard for studies carried out in organizational settings under the umbrella of positive psychology, as it would be the best way to ensure the prevention and reduction of pathological states and the achievement of high levels of adaptive functioning [6]. Although flourishing has been operationalized in research via various theoretical models [6,7,8], all of the models agree on the idea that this construct represents the combination of high levels of hedonic and eudaimonic well-being [9], which are the achievement of a pleasant, comfortable life (hedonia), as well as a full life that is consistent with the development of personal potential and the values which embody our true self (eudaimonia) [10]. Therefore, flourishing epitomises mental health as it is conceptualized today [11], since mental health not only implies the absence of mental illness, but also the presence of “a state of well-being in which the individual realizes his or her own abilities, can cope with the normal stresses of life, can work productively and fruitfully, and is able to make a contribution to his or her community” [12] (p. 10).

The prolific research activity around the identification of personal psychological resources that may best contribute to increased flourishing of workers in the organizational context has its highest expression in the construct of psychological capital (PsyCap) [13]. PsyCap is “an individual’s positive psychological state of development and is characterized by: (a) having confidence (efficacy) to take on and put in the necessary effort to succeed at challenging tasks; (b) making a positive attribution (optimism) about succeeding now and in the future; (c) persevering towards goals and, when necessary, redirecting paths to goals (hope) in order to succeed; and (d) when beset by problems and adversity, sustaining and bouncing back and even beyond (resiliency) to attain success” [14] (p. 2). PsyCap is therefore shaped by the interaction of the resources of efficacy, optimism, hope, and resilience, which implies that these four attributes work synergistically as a higher resource, such that their predictive ability would be significantly greater when taken together than when taken separately [15]. In this regard, various studies in occupational and academic environments have shown that PsyCap predicts levels of flourishing significantly better when it is considered as a global construct [16,17,18,19,20].

### 1.1. Psychological Capital in Teachers: Its Relationship to Flourishing and to Burnout

Although most studies about PsyCap have focused on organizational environments, such as business and healthcare, there has been growing attention paid in recent years to the teaching profession. One of the main reasons behind this unanticipated interest in psychological resources that encourage adaptive organizational functioning in teachers is the high levels of burnout that this group of workers suffer from [21,22,23]. Burnout is typically characterized by the clinical manifestation of three symptoms produced by the experience of chronic occupational stress: emotional exhaustion, depersonalization, and a lack of professional accomplishment [24,25]. Emotional exhaustion expresses itself through high levels of physical and mental tiredness, along with a lack of energy for facing the day to day demands of work, and a feeling of being overwhelmed by those demands. Depersonalization involves the development of attitudes of coldness, apathy, disinterest, and cynicism in workplace relationships (e.g., with students and other teachers). A lack of professional accomplishment is linked to a general feeling of stagnation, which is often accompanied by an intense desire to give up the profession.

Studies in the area of teacher burnout have been conclusive about the negative relationship between PsyCap and burnout [26,27,28,29,30], which suggests that PsyCap would be an effective personal resource for the reduction of these psychopathological states in teachers. This solid evidence is in contrast to the scant research about the contribution of PsyCap to teacher flourishing, which is consistent with the relatively young research tradition about aspects linked to well-being in this occupational group [31,32]. Within this fresh approach, attention has been mainly paid to hedonic aspects [33], finding that PsyCap encourages the experience of high levels of satisfaction and positive emotions in teaching [34,35]. In addition, some recent studies have documented PsyCap’s positive contribution to teachers’ eudaimonic well-being. In a longitudinal study with university teachers, Li [36] found that PsyCap was a long-term predictor (both directly and indirectly, via meaning in life) of psychological well-being, which is self-acceptance, positive relationships with others, autonomy, environmental mastery, purpose in life, and personal growth. Kurt and Demirbolat [37] concluded that PsyCap directly, and indirectly (via job satisfaction), improved eudaimonic well-being in secondary-school teachers.

### 1.2. The Current Study

**Figure 1:**
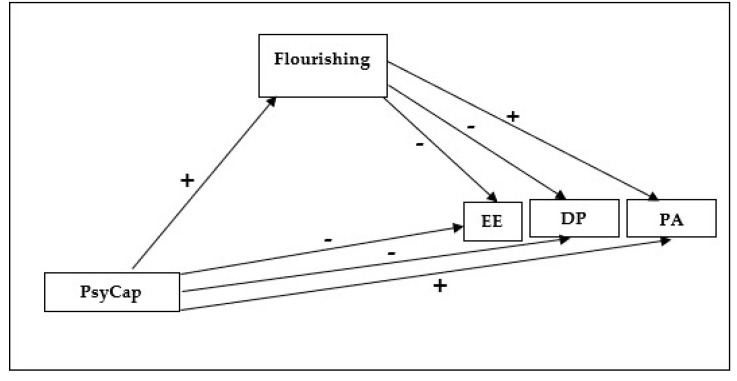
Hypothesized mediational model for the relationship between PsyCap, flourishing, and burnout. PsyCap = Psychological Capital; EE = Emotional Exhaustion; DP = Depersonalization; PA = Professional Accomplishment.

In line with the growing interest in aspects related to flourishing in teaching, the aim of this study is to examine the mediating role of flourishing in the relationship between PsyCap and the three burnout dimensions, namely emotional exhaustion, depersonalization, and a lack of professional accomplishment (see Figure 1).

Although the studies we reviewed above indicate that PsyCap contributes to experiencing high levels of hedonic and eudaimonic well-being, as well as reduced teacher burnout, to the best of our knowledge, there is no research that has examined the relationship between these three variables together. However, there are various reasons leading us to hypothesize that the negative effect of PsyCap on the burnout dimensions will be partly mediated by flourishing. According to the widely-accepted job-demands resources model (JD-R) [38], PsyCap is a personal psychological resource that—in highly demanding occupational contexts, which are therefore potentially harmful to physical and psychological health—would not only directly reduce the likelihood of experiencing burnout, but would also stimulate worker well-being (i.e., flourishing). In turn, the JD-R model states that occupational well-being would reduce the tendency to experience burnout, although it also posits that high levels of burnout would negatively affect a worker’s well-being [39]. This dual approach demonstrates the heuristic nature of the JD-R model, in light of which both flourishing and burnout may be mediators or outcomes [40].

In line with this lack of consensus about the role played by flourishing and burnout in their relationship with PsyCap, Manzano-García and Ayala [19] have shown a moderating effect of burnout between PsyCap and eudaimonic well-being in direct support staff of specialist autism services. In contrast, Polizzi Filho and Claro [41] found that PsyCap and hedonic well-being were negative predictors of turnover intention (i.e., an indicator of the lack of professional accomplishment dimension of burnout) in teachers.

The results from Polizzi Filho and Claro are therefore consistent with our hypothesis that flourishing has a negative (mediating) effect on teacher burnout. Although to date the negative relationship between flourishing and burnout has not been clearly demonstrated in teachers, various arguments lead us to expect this effect. First, studies such as Redelinghuys, Rothman, and Botha [42] have found high levels of flourishing in teachers to be related to a lower likelihood of leaving the profession, a factor that is associated with one of the burnout dimensions, a lack of professional accomplishment. Second, studies with other professions [43] have found an inverse relationship between flourishing and burnout. Third, the idea of flourishing as a negative predictor of teacher burnout links conceptually with the positive psychology approach, which emphasizes the idea that flourishing represents the mirror opposite of the symptoms of common mental disorders [9], and as such is the best guarantee of preventing or reducing these pathologies (e.g., burnout).

In light of these arguments, one might assume that teachers with high PsyCap will be better able to experience states of flourishing in the performance of their job, and both aspects (PsyCap and flourishing) would reduce vulnerability to burnout. This would be consistent with the approach of the theory of Conservation of Resources (COR) [44], which suggests that people with personal resources exhibit strong motivation to acquire, maintain, and develop new resources (e.g., PsyCap) when facing occupational demands, and this spiral of positive gains would result in long-term adaptive personal results (e.g., high levels of flourishing, low levels of burnout). In short, within the relatively recent interest in the study of aspects linked to positive psychological functioning in teachers, the main contribution of this study lies in examining the role of teacher flourishing, not only as a possible desirable effect of PsyCap, but also as a major contributor to the reduction of burnout in this professional group.

We have chosen to statistically control the effect of gender, educational stage, and teacher experience in our study because, as previous research has shown, these sociodemographic variables seem to differentially influence teachers’ experience of burnout. In terms of gender, some studies have indicated that burnout is more pronounced in women [45], whereas others have reported the opposite [46]. It is possible that men and women display themselves differently in their vulnerability to the various burnout symptoms. Women would be more likely to suffer emotional exhaustion while men would be more affected by depersonalization and a lack of professional accomplishment [47,48,49,50,51]. The educational stage and professional experience may also be significant factors in the explanation of burnout, with greater vulnerability to the syndrome in the higher educational stages [52,53] and in teachers with little experience [54,55].

## 2. Materials and Methods

### 2.1. Participants

A total of 1379 teachers from pre-school, primary, and secondary education, as well as vocational systems (1016 women, 363 men) from Galicia (Spain) took part in the study. They were aged between 24 and 63 years old (*M* = 43.17; *SD* = 13.21). The distribution by educational stage was as follows: 107 (7.8%) worked in pre-school education (students aged 3–6); 297 (21.5%) worked in primary education (ages 6–12); 472 (34.2%) worked in compulsory secondary education (ages 12–16); 127 (9.2%) taught Bachillerato (Baccalaureate) (ages 16–18); 11 taught vocational training; and 365 (26.5%) taught in more than one educational stage. In terms of experience, 6 (0.4%) participants had less than 1 year of teaching experience, 204 (14.8%) had between 2 and 5, 137 (9.9%) had between 5 and 10, 386 (28%) had between 10 and 20, 409 (29.7%) had between 20 and 30, and 237 (17.2%) had more than 30 years’ experience.

### 2.2. Instruments

Psychological capital: We used the CapPsi Psychological Capital Scale [28,56]. It consists of 16 items which evaluate the four resources (efficacy, hope, optimism, and resilience) which synergistically make up human psychological capital [14]. The responses are given on a six-point Likert-type scale (1 = completely disagree, to 6 = completely agree). Higher responses indicate a higher level of self-reported PsyCap. The internal consistency of the instrument in our study was α = 0.90, ω = 0.90 (95% CI [89,90]).

Flourishing: We assessed flourishing using the eight items making up the Flourishing Scale [57], Spanish version [58]. It uses a Likert-type response scale ranging from 1 (completely disagree) to 5 (completely agree). Higher scores indicate higher levels of flourishing. The internal consistency of the instrument was α = 0.88, ω = 0.88 (95% CI [0.87, 0.89]).

Burnout: We measured burnout using the Spanish adaptation [59] of the Maslach Burnout Inventory-Educators Survey (MBI-ES) [60]. It consists of 22 items that evaluate the three manifestations of the syndrome (emotional exhaustion, depersonalization, and lack of professional accomplishment). According to the original authors’ recommendations, each of these characteristics should be assessed individually. The emotional exhaustion scale has nine items (α = 0.90, ω = 0.91 (95% CI [0.89, 0.91]). The depersonalization scale has five items (α = 0.62, ω = 0.63 (95% CI [0.58, 0.64]). The professional accomplishment scale has eight items (α = 0.84, ω = 0.83 (95% CI [0.82, 0.85]). The responses to all of the items are given using a Likert-type scale with responses from 0 (never) to 6 (always). Higher scores indicate higher levels of emotional exhaustion and depersonalization, and lower scores indicate lower levels of professional accomplishment.

### 2.3. Procedure

We began the data collection process by sending emails to schools in the four provinces in Galicia. The email detailed the study objectives and the terms of participation (voluntary, anonymous, and confidential). It also included a link to an online platform which contained the measurement instruments items, the instructions for responding to them, and a declaration of informed consent, in accordance with the ethical principles of the University of A Coruña (UDC Ethical Code of Research 27 February 2019) and the Declaration of Helsinki. We asked the schools to distribute the information in the email to the teaching staff. Thus, the study included those teachers who, after accepting the above conditions, responded to the questions via the online platform, with no time limit.

### 2.4. Data Analysis

Our first step was to perform a preliminary analysis by calculating the descriptive statistics (means, standard deviations, asymmetry, and kurtosis) and (Pearson) correlation matrix for the study variables. Following that, in order to test our hypothesis, we performed a mediation analysis using the PROCESS macro in the IBM SPSS Statistics for Windows, version 22 (IBM Corp., Armonk, NY, USA) [61] statistics package. In line with the hypothesized mediation model (Figure 1), we specified PsyCap as the independent variable and the three burnout dimensions (emotional exhaustion, depersonalization, and lack of professional accomplishment) as dependent variables in the model. We added flourishing as a mediating variable of the effect of PsyCap on the three burnout dimensions. In addition, we specified gender, educational stage, and teacher experience as covariables in order to statistically control for their effect on the three burnout dimensions. The effect size was determined using Cohen’s *d* [62]: null: *d* < 0.09; small: *d* = 0.10—*d* = 0.49; medium: *d* = 0.50—*d* = 0.79; and large: *d* ≥ 0.80.

## 3. Results

### 3.1. Descriptive and Correlation Analysis

Table 1 shows the descriptive statistics and correlations for the variables. The data for asymmetry and kurtosis indicate that the variables exhibited a normal distribution. In addition, all of the variables demonstrated statistically significant correlations (*p* < 0.001). Both PsyCap and flourishing were negatively correlated with emotional exhaustion (*r* = −0.43, *p* < 0.001; and *r* = −0.38, *p* < 0.001, respectively) and depersonalization (*r* = −31, *p* < 0.001 with psychological capital and *r* = −0.33, *p* < 0.001 with flourishing). In contrast, the correlations with professional accomplishment were positive. We also found a positive correlation between PsyCap and flourishing (*r* = 0.66, *p* < 0.001). These indicators supported the suitability of subsequent multivariate analysis.

### 3.2. Mediation Analysis

The mediation effect of flourishing was assessed using the bootstrap estimation procedure. Following the recommendations of MacKinnon et al. [63], we used a bootstrap sample of 5000 cases and a confidence interval of 95%, given that, in general, estimations of indirect effects do not follow a normal distribution. Table 2 presents the direct, indirect, and total effects of the mediation analysis in the relationship between PsyCap and emotional exhaustion.

The data confirmed our initial hypotheses, as once the effects of covariables (gender, educational stage, and teacher experience) were statistically controlled for, flourishing partially mediated the effect of PsyCap on emotional exhaustion. More specifically, PsyCap exercised a significant direct effect on both flourishing and emotional exhaustion. For flourishing, this effect was positive (*b* = 0.727; *p* < 0.001), with a large effect size (*d* = 3.55), whereas the effect on emotional exhaustion was negative (*b* = −0.767; *p* < 0.001), with a moderate effect size (*d* = 0.56). Flourishing in turn had a significant, direct, negative effect on emotional exhaustion (*b* = −0.372; *p* < 0.001), albeit with a small effect size (*d* = 0.29). We also found a significant indirect effect of PsyCap on emotional exhaustion via flourishing (*b* = –0.271; 95% CI [−0.309, −0.113]). The confidence intervals do not include zero, indicating that this indirect effect was statistically significant. The overall effect of the model was also statistically significant (*b* = –1.038; *p* < 0.001), with a large effect size (*d* = 1.09). With regard to the effects of the covariables, we found that they had a significant effect on both flourishing (*b* = −0.063; *p* < 0.05) and emotional exhaustion (*b* = –0.164; *p* < 0.05), although with a small effect size (*d* = 0.12 in both cases). Being a woman predicted a greater level of flourishing and emotional exhaustion. The effects of the other two covariables (educational stage and experience) were not statistically significant.

We also confirmed the hypothesized effect of flourishing’s partial mediation in the relationship between PsyCap and depersonalization, once the effect of the three covariables was controlled for. As Table 3 shows, PsyCap had a significant negative effect on depersonalization, both directly (*b* = –0.274; *p* < 0.001; *d* = 0.27) and indirectly through flourishing (*b* = –0.244; 95% CI [–0.337, –0.153]). We also found a significant direct negative effect of flourishing on depersonalization (*b* = –0.335; *p* < 0.001), with a small effect size (*d* = 0.36). The total effect of the model was also statistically significant (*b* = –0.518; *p* < 0.001), with a moderate effect size (*d* = 0.69). With regard to the effects of the covariables, gender (*b* = 0.260; *p* < 0.001), educational stage (*b* = 0.027; *p* < 0.05), and teacher experience (*b* = –0.064; *p* < 0.001) were significant predictors of depersonalization, albeit with small effect sizes (*d* < 0.30, in each case). Being a man, teaching in a higher educational stage, and having less experience predicted greater levels of depersonalization.

Lastly, the data also supported our initial hypothesis about the partial mediating effect of flourishing in the relationship of PsyCap and the lack of professional accomplishment. As the results in Table 4 show, once the effect of the covariables was statistically controlled for, we found that PsyCap had a significant positive effect on professional accomplishment, both directly (*b* = 1.005; *p* < 0.001; large effect size, *d* = 1.61) and indirectly through flourishing (*b* = 0.140; 95% CI [0.079, 0.209]). Flourishing was also shown to be a significant positive predictor of professional accomplishment (*b* = 0.193; *p* < 0.001), although the effect size was small (*d* = 0.27). The total effect of the model was also statistically significant (*b* = 1.145; *p* < 0.001), with a large effect size (*d* = 5.53). With regard to the covariables, only the educational stage demonstrated a statistically significant effect, which was negative, on professional accomplishment (*b* = –0.028; *p* < 0.05), with a small effect size (*d* = 0.15).

## 4. Discussion

Since flourishing has been considered a key objective for the development of healthy organizational environments, there has been growing interest in the identification of personal psychological resources that encourage workers’ successful adaptation to the complex, changing demands of the modern workplace [2]. In line with this proactive positioning, in this study, we examined the effect of PsyCap and flourishing on burnout in teachers, given its high prevalence in this professional group [21,22,23]. Specifically, we explored the possible mediating role of flourishing in the relationship between PsyCap and each of the symptomatic manifestations of burnout (emotional exhaustion, depersonalization, and lack of professional accomplishment).

Our results confirmed our initial hypothesis. They showed that PsyCap has a direct effect on teacher flourishing and on the three burnout indicators. The effect of PsyCap on flourishing was positive, indicating that the availability of high levels of efficacy, optimism, hope, and resilience in teaching is related to experiencing high levels of hedonic and eudaimonic well-being. This finding seems to align with recent research focused on teacher well-being [31,32], underlining the significant contribution of PsyCap as a personal resource that enhances the lives of teachers, not only in terms of satisfaction, pleasure, and enjoyment, but also as a means of attaining high levels of personal excellence, as other studies have concluded [34,35,36,37]. In addition, the positive effect of PsyCap on flourishing would confirm, along the lines laid out by the JD-R model [39], the significant contribution made by personal resources to well-being in occupational settings. More specifically, this positive impact on well-being was produced directly, which is consistent with other studies on teachers [36,64].

In contrast, our data show that the direct effect of PsyCap on the three characteristic burnout sypmtoms was negative. This suggests, in line with other studies [26,27,28,29,30], that PsyCap—considered as a paradigmatic example of positive organizational behavior—plays a significant role when it comes to reducing teachers’ vulnerability to feeling burned out in their day to day professional experience.

The negative effect of PsyCap on burnout is not only direct. Based on the results of our study the relationship is also partially mediated by flourishing. One notable implication resulting from this finding is the fact that flourishing, in addition to being an optimal outcome of the effective use of PsyCap in teaching, is also in itself an adaptive personal resource in the reduction of the three clinical manifestations of burnout. In this regard, studies such as Redelinghuys et al. [42] have already noted the negative effect of flourishing on one of the main indicators of a lack of professional accomplishment, the intention to quit the teaching profession. In addition to supporting this relationship, our data demonstrate that flourishing also contributes to reducing the tendency to experience the two “core” manifestations of burnout [65] (emotional exhaustion and depersonalization).

The consideration of flourishing as a personal resource promoted by PsyCap would also be consistent with the approach of COR theory [44], in that one may assume that teachers who have a high level of psychological resources (efficacy, optimism, hope, and resilience) would be involved in a rising spiral of acquisition, development, and preservation of new resources (i.e., indicators of flourishing such as life satisfaction, positive affect, purpose in life, autonomy, and relatedness). In contrast, the absence of PsyCap would bring with it the loss of new resources (flourishing) to face the demands of work, which would contribute to experiencing pathological states (burnout).

In effect, as the JD-R model postulates [38], suffering from work-related burnout usually occurrs in the context of high work-related demands and scarce resources to cope with them. For teachers, the need to respond to a growing number and variety of educational and bureaucratic demands, the lack of student motivation and issues of student discipline, and a lack of institutional support [66,67], together with a lack of psychological resources (e.g., low PsyCap, deficient skills for emotional regulation and stress management, personality with neurotic tendencies) [68,69,70,71], make up a breeding ground in which burnout may develop. Nevertheless, in accordance with the model, even in highly demanding situations, the availability of personal resources would significantly reduce vulnerability to stress and burnout [72].

The results of our study seem to confirm this thesis. It is not for nothing that those teachers who exhibit high levels of self-confidence in their capacity to successfully deal with professional challenges (efficacy), to perservere and manage any eventual obstacle in the achievement of a goal (hope), to resist and come through adversity stronger (resilience), and to maintain a positive outlook about their present and future work (optimism), are the teachers who have a range of personal resources that make them less prone to feeling physically and emotionally exhausted, cold and distant in their interpersonal relationships, or lacking accomplishment in their work. Instead, these resources provide teachers with sound personal capital that will lead them to experience high levels of hedonic and eudaimonic well-being in their day to day work.

### 4.1. Implications for Intervention

We believe that the findings from our study may be an important contribution to the growing demand for effective interventions aimed at improving the psychological health of workers and organizations as a whole [2]. Our results show that both PsyCap and states of flourishing have a significant negative effect on the three symptomatic manifestations of burnout. This finding points to the possiblity that both factors (PsyCap and flourishing) may be effective psychological tools in the reduction of burnout symptoms in teachers. Given that, and the fact that both PsyCap and flourishing are considered to have a state-like, developable rather than dispositional, nature [15,73], the design of interventions aimed at stimulating teacher PsyCap and flourishing may be one mode of action to take in order to prevent the occurrence of health problems and encourage optimal positive functioning in this professional group.

There has been a proliferation of interventions in recent years aimed at the development of PsyCap and flourishing in a wide range of occupational settings [74,75], although only a few initiatives centered on PsyCap have focused on teachers [76,77]. For PsyCap these interventions have often consisted in micro-interventions (four- to eight weeks of sessions, each lasting between one and four hours) in which, among other things, teachers learn to set goals (specific, realistic, consistent with personal values and challenges), design alternative plans in case of obstacles, develop positive self-dialogs, visualize experiences of success, identify resources, and reduce risks, as well as implementing adaptive social skills when it comes to dealing with problems. Interventions aimed at stimulating flourishing have demonstrated a great variety of content, although initiatives aimed at encouraging gratitude, contribution to the community, work engagement, and social connectedness have been effective in workers in various professional environments [74].

To date, most interventions in the organizational context have not considered the combination of PsyCap and flourishing. However, the results of this study seem to indicate that this type of initiative would be more beneficial if both factors were worked on in combination, making use of the caravan action that, according to COR theory [78], is produced when new personal psychological resources are acquired and developed.

### 4.2. Study Limitations and Future Directions

The contributions of this study must be considered in light of the study’s limitations. Firstly, the transversal design of the study does not allow us to establish causal relationships between PsyCap, flourishing, and burnout. In this regard, experimental or longitudinal studies are needed which will allow us to determine whether an intervention focused on encouraging teacher PsyCap would produce improvements in their levels of flourishing and whether this would in turn result in them being less prone to experiencing emotional exhaustion, depersonalization, or a lack of professional accomplishment. Secondly, the use of self-report scales as the only data collection procedure may bring with it some biases (for example, social desirability). Future studies should therefore test the findings of our study by referring to other complementary sources for the collection of information (in-depth interviews, observations, etc.). In addition, the use of a convenience sample is a limitation in terms of obtaining a balanced sample of teachers with regard to variables such as gender, educational stage, or professional experience. To the extent that this sampling procedure does not guarantee representativeness of the teaching population, this may result in a bias in the results. New studies are needed which use more rigorous sampling procedures in order to make it possible to generalize the findings to the teaching population as a whole.

In this study, we considered three variables (gender, educational stage, and professional experience) as covariables in order to statistically control for their effect. Our data indicate that some of them may play a significant role (although small in terms of effect size) in the experience of teacher burnout. More specifically, women seem to be more prone to emotional exhaustion and men more prone to depersonalization. Both depersonalization and the lack of professional accomplishment seem to be more pronounced the higher the educational stage being taught. This is consistent with findings from other studies [47,48,49,50,51,52,53], and it would be advisable to take them into consideration when designing interventions aimed at preventing or reducing burnout in teachers.

## 5. Conclusions

Flourishing has been considered as a “promising new outcome variable for POB (positive organizational behavior) researchers” [79] (p. 50). In line with this new paradigm focused on the promotion of health in the organizational context, the main contribution of this study was the identification of flourishing as a mediating variable in the relationship between PsyCap and the symptoms of burnout in teachers, a group that faces high occupational demands. These findings indicate that flourishing is not only a desirable effect of the availability of a high level of PsyCap, but that it is also a valuable psychological resource supporting PsyCap, contributing to a reduced likelihood of suffering from burnout. Consequently, interventions designed to enhance teachers’ psychological health and that of schools overall should consider the possible synergistic action of PsyCap and flourishing to promote initiatives aimed at the development of both personal resources.

## Figures and Tables

**Table 1:** Correlation matrix and descriptive statistics (mean, standard deviation, asymmetry, and kurtosis) for the study variables.

	1	2	3	4	5
Psychological Capital	−				
2. Flourishing	0.66 *	−			
3. Emotional exhaustion	−0.43 *	−0.38 *	−		
4. Depersonalization	−0.31 *	−0.33 *	0.41 *	−	
5. Professional accomplishment	0.68 *	0.53 *	−0.42 *	−0.41 *	−
*M*	4.00	4.22	2.35	0.91	4.39
*SD*	0.53	0.59	1.28	0.91	0.89
*Asymmetry*	−0.77	−1.00	0.51	1.31	−0.48
*Kurtosis*	1.48	1.77	−0.39	1.90	0.23

Note: PsyCap scale (1–6), with higher scores indicating a higher level of PsyCap. Flourishing scale (1–5), with higher scores indicating a higher level of flourishing. Burnout scales (0–6), with higher scores indicating greater levels of emotional exhaustion and depersonalization, and lower scores indicating lower levels of professional accomplishment; * *p* < 0.001.

**Table 2:** Results of the mediation analysis of flourishing in the relationship between PsyCap and emotional exhaustion.

Description of the Model	Coef.	SE	*t*	*p*	*d*	LCI	UCI
**Direct effect**							
PCA→FLO	0.727	0.022	32.365	0.000	3.55	0.683	0.771
GEN→FLO	−0.063	0.027	–2.303	0.021	0.12	–0.116	–0.009
STA→FLO	–0.010	0.007	–1.436	0.151	—	–0.024	0.003
EXP→FLO	–0.004	0.009	–0.527	0.598	—	–0.023	0.013
**Direct effect**							
PCA→EME	–0.767	0.076	–9.978	0.000	0.56	–0.917	–0.616
FLO→EME	–0.372	0.069	–5.360	0.000	0.29	–0.509	–0.236
GEN→EME	–0.164	0.070	–2.322	0.020	0.12	–0.302	–0.025
STA→EME	0.033	0.018	1.846	0.065	—	–0.002	0.069
EXP→EME	0.036	0.023	1.540	0.123	—	–0.010	0.083
**Indirect effect**							
PCA→FLO→EME	–0.271	0.064	—	—	—	–0.309	–0.113
**Total effect**	–1.038	0.058	–17.749	0.000	1.09	–1.152	–0.923

Note: PCA = Psychological capital; FLO = Flourishing; EME = Emotional exhaustion; GEN = Gender; STA = Educational stage; EXP = Teacher experience. PsyCap scale (1–6), with higher scores indicating a higher level of PsyCap. Flourishing scale (1–5), with higher scores indicating a higher level of flourishing. Emotional exhaustion scale (0–6), with higher scores indicating greater levels of emotional exhaustion; Gender: 1 = Woman, 2 = Man; LCI = Lower confidence interval (95%); UCI = Upper confidence interval (95%).

**Table 3:** Results of the mediation analysis of flourishing in the relationship between PsyCap and depersonalization.

Description of the Model	Coef.	SE	*t*	*p*	*d*	LCI	UCI
**Direct effect**							
PCA→DEP	–0.274	0.056	–4.877	0.000	0.27	–0.384	–0.164
FLO→DEP	–0.335	0.059	–6.596	0.000	0.36	–0.435	–0.236
GEN→DEP	0.260	0.051	5.040	0.000	0.27	0.159	0.362
STA→DEP	0.027	0.013	2.029	0.042	0.11	0.001	0.053
EXP→DEP	–0.064	0.017	–3.654	0.000	0.20	–0.098	–0.029
**Indirect effect**							
PCA→FLO→DEP	–0.244	0.046	—	—	—	–0.337	–0.153
**Total effect**	–0.518	0.043	–12.050	0.000	0.69	–0.603	–0.434

Note: PCA = Psychological capital; FLO = Flourishing; DEP = Depersonalization; GEN = Gender; STA = Educational stage; EXP = Teacher experience. PsyCap scale (1–6), with higher scores indicating a higher level of PsyCap. Flourishing scale (1–5), with higher scores indicating a higher level of flourishing. Depersonalization scale (0–6), with higher scores indicating greater levels of Depersonalization; Gender: 1 = Woman, 2 = Man; LCI = Lower confidence interval (95%); UCI = Upper confidence interval (95%).

**Table 4:** Results of the mediation analysis of flourishing in the relationship between PsyCap and professional accomplishment.

Description of the Model	Coef.	SE	*t*	*p*	*d*	LCI	UCI
**Direct effect**							
PCA→PRO	1.005	0.043	23.272	0.000	1.61	0.920	1.089
FLO→PRO	0.193	0.039	4.956	0.000	0.27	0.117	0.270
GEN→PRO	–0.058	0.039	–1.485	0.137	—	–0.136	0.018
STA→PRO	–0.028	0.010	–2.790	0.005	0.15	–0.048	–0.008
EXP → PRO	0.005	0.013	0.437	0.662	—	–0.020	0.032
**Indirect effect**							
CAP → FLO → PRO	0.140	0.033	—	—	—	0.079	0.209
**Total effect**	1.145	0.032	34.924	0.000	5.53	1.081	1.210

Note: PCE = Psychological capital; FLO = Flourishing; PRO = Professional accomplishment; GEN = Gender; STA = Educational stage; EXP = Teacher experience. PsyCap scale (1–6), with higher scores indicating a higher level of PsyCap. Flourishing scale (1–5), with higher scores indicating a higher level of flourishing. Professional accomplishment scale (0–6), with lower scores indicating lower levels of professional accomplishment. Gender: 1 = Woman, 2 = Man; LCI = Lower confidence interval (95%); UCI = Upper confidence interval (95%).
